# Supplementary material for: The native ORAI channel trio underlies the diversity of Ca2+ signaling events
Source: Nat Commun. 2020 May 15;11:2444. doi: 10.1038/s41467-020-16232-6 (PMC7229178; doi:10.1038/s41467-020-16232-6)

The *Native* ORAI Channel Trio Underlies the Diversity of Ca^{2+} Signaling Events

By

Ryan E. Yoast[*,1], Scott M. Emrich[*,1], Xuexin Zhang[1], Ping Xin[1], Martin T. Johnson[1], Adam J. Fike[1], Vonn Walter[3,4,5], Nadine Hempel[2,5], David I. Yule[6], James Sneyd[7], Donald L. Gill[1], and Mohamed Trebak[#,1,5]

From

[1]Department of Cellular and Molecular Physiology, [2]Department of Pharmacology, [3]Department of Public Health Sciences, [4]Biochemistry and Molecular Biology, and [5]Penn State Cancer Institute, the Pennsylvania State University College of Medicine; 500 University Dr. Hershey, PA, 17033 USA. [6]Department of Pharmacology and Physiology, University of Rochester, Rochester, NY 14642 USA. [7]Department of Mathematics, The University of Auckland, 38 Princes Street, Auckland, 1010, New Zealand.

[*]Equal contribution

[#]Correspondence and requests for materials should be addressed to M.T. (email: mtrebak@psu.edu)

Supplementary Table 1. Key resources and reagents used in study

Reagent or resource	Source	Identifier
Antibodies		
Rabbit polyclonal anti-ORAI1 (1:1000)	MilliporeSigma	#O8264
Mouse monoclonal anti-GAPDH (1:5000) Clone 6C5	MilliporeSigma	#MAB374
STIM1 Antibody (1:2000)	Cell Signaling	#4916S
STIM2 Antibody (1:1000)	Cell Signaling	#4917S
α-IP3R1 (1:500)	Yule lab	NA
α-IP3R2 (1:500)	Yule lab	NA
Anti-IP3R-3 (1:1000) Clone 2/IP3R-3	Becton, Dickinson (BD)	#610313
α-Tubulin (DM1A) Mouse mAb (1:2000) Clone DM1A	Cell Signaling	#3873S
IRDye® 800CW Donkey anti-Rabbit IgG Secondary Antibody (1:10,000)	LI-COR	#926-32213

IRDye® 680RD Goat anti-Mouse IgG Secondary Antibody (1:10,000)	LI-COR	#926-68070
Chemicals		
Thapsigargin	Calbiochem	#586005
Carbachol	Sigma-Aldrich	#212385-100MG-M
Ionomycin (free acid)	AdipoGen	#AG-CN2-0416-M005
Fura-2 AM	Invitrogen	# F1201
BAPTA-tetracesium Salt	Santa Cruz Biotechnology	#480436-84-8
2-Aminoethoxydiphenyl borate (2-APB)	MilliporeSigma	#D9754-10G
PNGase F	Sigma-Aldrich	#11365169001
TRIzol	Invitrogen	#15596026
Cyclopiazonic Acid	Alomone labs	# C-750
Puromycin Dihydrochloride	MP Biomedicals	#ICN19453980
Trypan Blue Solution, 0.4%	Gibco	#15-250-061
Intercept® (TBS) Blocking Buffer	LI-COR	#927-60001
Halt Protease and Phosphatase Inhibitor	Thermo Scientific	#PI78443
Gadolinium(III) Chloride	ACROS Organics	#AC383560050
RIPA Buffer	Sigma-Aldrich	#R0278-50ML
**GSK-7975A**	Sigma-Aldrich	#534351
Oligonucleotides		
AllStars Control	Qiagen	Cat#1027281
siORAI1	Integrated DNA Technologies (IDT)	NA
Cell Lines		
HEK293 cells	ATCC	CRL-1573

ORAI1-KO cells	Trebak Lab[1]	NA
ORAI2-KO cells	Trebak Lab; This paper	NA
ORAI3-KO cells	Trebak Lab; This paper	NA
ORAI1,2-DKO cells	Trebak Lab; This paper	NA
ORAI1,3-DKO cells	Trebak Lab; This paper	NA
ORAI2,3-DKO cells	Trebak Lab; This paper	NA
ORAI-TKO cells	Trebak Lab; This paper	NA
Software		
Graphpad Prism 8	GraphPad	https://www.graphpad.com/scientific-software/prism/
Origin 9.0	OriginLab	https://www.originlab.com/
Clampfit 10.3	Molecular Devices, LLC.	https://www.moleculardevices.com/
ImageJ	Schneider et al., 2012	https://imagej.nih.gov/ij/
LAS X	Leica	https://www.leica-microsystems.com/
SlideBook 6.0	Intelligent Imaging Innovations, Inc.	https://www.intelligent-imaging.com/slidebook
MAXCHELATOR	Chris Patton: cpatton@stanford.edu	http://maxchelator.stanford.edu/
Image Studio Lite	LI-COR	https://www.licor.com/bio/image-studio-lite/download
Commercial Kits/Assays		
Guide-it Mutation Detection Kit	Clontech Laboratories	#631443
Cell line Nucleofector Kit	Lonza	#VCA-1003
In-Fusion® HD EcoDry™ Cloning System	Clontech Laboratories	#639688
DNase I	Invitrogen	#18068-15
High Capacity cDNA Reverse Transcription Kit	Applied Biosystems	#4368814
PowerUp SYBR Green Master Mix	Applied Biosystems	#100029285
Pierce™ Rapid Gold BCA Protein Assay Kit	Thermo Scientific	# A53225

Recombinant DNA		
HA-NFAT1(4-460)-GFP	Addgene	#11107
HA-NFAT4(3-407)-GFP	Addgene	#21664
LentiCRISPR v2	Addgene	#52961
pU6-(Bbs1)-mCherry	Addgene	#64324
pSpCas9(BB)-2A-GFP (PX458)	Addgene	#48138
pCMV G-CEPIA1er	Addgene	#58215
CMV-YFP-ORAI1	Trebak Lab; This paper	NA
CMV-YFP-ORAI2	Trebak Lab; This paper	NA
CMV-YFP-ORAI3	Trebak Lab; This paper	NA
CMV-CFP-ORAI1	Putney Lab	NA
CMV-CFP-ORAI2	Putney Lab	NA
CMV-CFP-ORAI3	Putney Lab	NA
TK-YFP-ORAI1	Trebak Lab; This paper	NA
TK-YFP-ORAI2	Trebak Lab; This paper	NA
TK-YFP-ORAI3	Trebak Lab; This paper	NA
CMV-STIM1-YFP	Trebak Lab; This paper	NA
CMV-STIM1-mCherry	Gill lab	NA
CMV-ORAI1-ORAI1-tdTomato	Gill lab	NA
CMV-ORAI1-ORAI2-tdTomato	Gill lab	NA
CMV-ORAI1-ORAI3-tdTomato	Gill lab	NA
CMV-ORAI2-ORAI3-tdTomato	Trebak Lab; This paper	NA
CMV-CFP-ORAI1 (E106Q)	Gill lab	NA
CMV-CFP-ORAI1 (E106A)	Gill lab	NA

CMV-CFP-ORAI2 (E80Q)	Trebak Lab; This paper	NA
CMV-CFP-ORAI3 (E81Q)	Trebak Lab; This paper	NA

Supplementary Table 2. Oligonucleotide sequences used for RT-qPCR and cloning

**qPCR Primers**		
Primers	Forward 5’→3’	Reverse 5’→3’
ORAI1	GATGAGCCTCAACGAGCACT	ATTGCCACCATGGCGAAGC
ORAI2	TGGCGGAAGCTCTACCTGAG	CGGGTACTGGTACTGCGTC
ORAI3	GAGTGACCACGAGTACCCACC	GGGTACCATGATGGCTGTGG
GAPDH	CCCTTCATTGACCTCAACTACA	ATGACAAGCTTCCCGTTCTC
**Cloning Primers**		
CMV-ORAI2-ORAI3-tdTomato	GTTGGTAAAGCCACCATGAGTGCTGAGCTTAACGTG	AAAGATCCTCTAGAACTACTTGTACAGCTCGTCCAT
ORAI2 (E80Q) mutation	CCATGGTGCAGGTGCAGCTGGAGACGC	GCACCTGCACCATGGCCACCATGGC
ORAI3 (E81Q) mutation	CCATGGTGCAGGTGCAGCTGGAGAGTGACC	GCACCTGCACCATGGCCACCATGGC
ORAI1-KO CRISPR/Cas9 gRNA	CACCGGTTGCTCACCGCCTCGATGT	AAACACATCGAGGCGGTGAGCAACC
ORAI2-KO CRISPR/Cas9 gRNA #1	CACCGACGACAGGGCCTGTACCGAG	AAACCTCGGTACAGGCCCTGTCGTC
ORAI2-KO CRISPR/Cas9 gRNA #2	CACCGCTCATGCGGGGACTCGCTGA	AAACTCAGCGAGTCCCCGCATGAGC
ORAI3-KO CRISPR/Cas9 gRNA #1	CACCGGTTCGTGCACCGCGGCTACC	AAACGGTAGCCGCGGTGCACGAACC
ORAI3-KO CRISPR/Cas9 gRNA #2	CACCGCCAGAGACTGCACCGCTACG	AAACCGTAGCGGTGCAGTCTCTGGC
To remove tk-promotor from vector	GGCTAGCGCTACCGGTCGCCA	CTCATTTAGACCCCGTAATTGATTACTA
To insert tk-promotor in front of all ORAI isoforms	GGCTAGCGCTACCGGTCGCCA	CCGGTAGCGCTAGCCTATAGTGAGTCGTATT

Supplementary Table 3. List of siRNA sequences

siRNA	**Sequence**	**Source**
AllStars Control	Commercial product (N/A)	Qiagen

siORAI1	CGUGCACAAUCUCAACUC GUU	Integrated DNA Technologies (IDT)

Supplementary Table 4. **Sequencing results validating the genetic knockout of all ORAI isoforms**

Knockout Cell Line:	CRISPR gRNA:	Description:
**ORAI1-SKO Clone #1**	ORAI1 g1: GTTGCTCACCGCCTCGATGT	Allele 1: Two nucleotides were deleted at positions 440-441 resulting in a frameshift of all following nucleotides.<br>Allele 2: A 40 nucleotide deletion from nucleotides 443-482 resulted in a frameshift mutation. All amino acids from position 148 on are out of frame. This resulted in a stop codon at amino acid 175.
**ORAI1-SKO Clone #9**	ORAI1 g1: GTTGCTCACCGCCTCGATGT	Allele 1: The deletion of the 16 nucleotides at positions 427-442 resulted in a frameshift.<br>Allele 2: The deletion of the 11 nucleotides at positions 432-442 resulted in a frameshift mutation.
**ORAI2-SKO Clone #5**	ORAI2 g1: ACGACAGGGCCTGTACCGAG<br>ORAI2 g3: CTCATGCGGGGACTCGCTGA	Allele 1 and 2: An identical change was observed for both ORAI2 alleles in this CRISPR clone. A 272 bp nucleotide deletion spanning nucleotides 132-403 resulted in a frameshift mutation.
**ORAI2-SKO Clone #22**	ORAI2 g1: ACGACAGGGCCTGTACCGAG.<br>ORAI2 g3: CTCATGCGGGGACTCGCTGA	Allele 1: A 272bp deletion spanning nucleotides 132-403 in a frameshift mutation and the generation of an early stop codon 4 amino acids after the first cut site.

		Allele 2: A 271bp deletion spanning nucleotides 133-403 knocked ORAI2 out of frame from amino acid 45 on.
**ORAI3-SKO Clone #25**	ORAI3 g2: GTTCGTGCACCGCGGCTACC	Allele 1: Insertion of 183 nucleotides that results in a stop codon after the 34th amino acid.<br>Allele 2: Deletion of nucleotide 310 (A) that results in a frame shift.
**ORAI3-SKO Clone #9**	ORAI3 g2: GTTCGTGCACCGCGGCTACC<br>ORAI3 g4: CCAGAGACTGCACCGCTACG	Allele 1 and 2: There was an identical 332 nucleotide deletion that spans nucleotide 104-436. This resulted in a frameshift from amino acid 35 on resulting in a stop codon at amino acid 54.
**ORAI2,3-DKO Clone #22**	ORAI2 g1: ACGACAGGGCCTGTACCGAG.<br>ORAI2 g3: CTCATGCGGGGACTCGCTGA	Parental cell line: ORAI3-SKO Clone #25<br>ORAI2KO, large genomic deletion was resolved by PCR see *supplementary figure 2C*.<br>Allele 1: There was a 272 bp deletion spanning nucleotides 132-403. In place of this 272bp deletion there was a 193bp insertion. These changes knocked ORAI2 out of frame.<br>Allele 2: There was a deletion ranging from nucleotide 132-403, this results in a 272 bp deletion.
**ORAI2,3-DKO Clone #9**	ORAI2 g1: ACGACAGGGCCTGTACCGAG.<br>ORAI2 g3: CTCATGCGGGGACTCGCTGA	Parental cell line: ORAI3-SKO Clone #25<br>Allele 1 and 2: There was a 272 nucleotide deletion spanning nucleotides 132-403. In place of this 272bp deletion there was a 163bp insertion. These changes

		knocked ORAI2 out of frame.
**ORAI1,3-DKO Clone #3**	ORAI3 g2: GTTCGTGCACCGCGGCTACC<br>ORAI3 g4: CCAGAGACTGCACCGCTACG	Parental cell line: ORAI1-SKO #6<br>Allele 1: A 332 nucleotide deletion removed nucleotides 104-435 resulting in a frameshift mutation.<br>Allele 2: A 308 nucleotide deletion removed nucleotides 104-435. At the cut site 308 nucleotides were inserted resulting in a frameshift mutation.
**ORAI1,3-DKO Clone #48**	ORAI3 g2: GTTCGTGCACCGCGGCTACC<br>ORAI3 g4: CCAGAGACTGCACCGCTACG	Parental cell line: ORAI1-SKO #1<br>Allele 1: A 332 nucleotide deletion removed nucleotides 104-435. There was a single nucleotide (T) added after the first cut site. This resulted in a frameshift after the cut site of the first gRNA. All amino acids after amino acid 35 are out of frame.<br>Allele 2: The random insertion after the first gRNA cut site resulted in a protein that is out of frame from 34 amino acid on.
**ORAI1,2-DKO Clone #18**	ORAI2 g1: ACGACAGGGCCTGTACCGAG.<br>ORAI2 g3: CTCATGCGGGGACTCGCTGA	Parental cell line: ORAI1-SKO #6.<br>Allele 1 and 2 are the same: A deletion of 271 nucleotides (133-403) resulted in a frameshift from amino acid 45 on.
**ORAI1,2-DKO Clone #19**	ORAI2 g1: ACGACAGGGCCTGTACCGAG<br>ORAI2 g3: CTCATGCGGGGACTCGCTGA	Parental cell line: ORAI1-SKO #1.<br>ORAI2:<br>Allele 1: A large deletion removed 337 nucleotides (132-468), this change

		resulted in a frameshift from amino acid 45 on.<br>Allele 2: Large genomic deletion of 271 nucleotides spanning 134-404. This results in a frameshift mutation from amino acid 45 until the end of the protein.
**ORAI-TKO #47**	ORAI3 g2: GTTCGTGCACCGCGGCTACC<br>ORAI3 g4:<br>CCAGAGACTGCACCGCTACG	Parental cell line:<br>ORAI1,2-DKO #19<br>Allele 1 and 2: A 235 nucleotide deletion removed nucleotides 104-435. This resulted in a frameshift from amino acid 35 on. This also caused a nonsense stop codon on amino acid 54.
**ORAI-TKO #53**	ORAI3 g2: GTTCGTGCACCGCGGCTACC<br>ORAI3 g4:<br>CCAGAGACTGCACCGCTACG	Parental cell line:<br>ORAI1,2-DKO #19<br>Allele 1: A deletion of 103 nucleotides removed nucleotides 104-435 and resulted in the removal of amino acid 36-146. These amino acids span transmembrane regions 1, 2, and part of transmembrane 3.<br>Allele 2: There was an insertion after the first gRNA cut site, nucleotide 103 that resulted in an insertion and frameshift from amino acid 35 on.

Note: All nucleotide annotations are referenced to the coding sequence of the corresponding ORAI mRNA. We excluded the 5’ and 3’ untranslated regions when aligning our sequencing results. (ORAI1: NM_032790, ORAI2: NM_001126340.3, and ORAI3: NM_152288.3).

Supplementary Table 5. mRNA expression of ORAI isoforms in ORAI-SKO and ORAI-DKO cell lines (normalized to parental HEK293 cells).

Cell Line	ORAI1	ORAI2	ORAI3

**Parental HEK293**	1	1	1
**ORAI1-SKO Clone #1**	NA	0.918±0.037	0.835±0.033
**ORAI1-SKO Clone #6**	NA	0.869±0.004	0.742±0.009
**ORAI2-SKO Clone #5**	1.004±0.055	NA	2.041±0.097
**ORAI2-SKO Clone #22**	1.300±0.029	NA	1.630±0.138
**ORAI3-SKO Clone #9**	1.440±0.040	1.200±0.062	NA
**ORAI3-SKO Clone #25**	1.035±0.024	0.980±0.031	NA
**ORAI2,3-DKO Clone #9**	0.972±0.025	NA	NA
**ORAI2,3-DKO Clone #22**	0.952±0.032	NA	NA
**ORAI1,3-DKO Clone #3**	NA	0.822±0.038	NA
**ORAI1,3-DKO Clone #48**	NA	0.610±0.022	NA
**ORAI1,2-DKO Clone #18**	NA	NA	3.081±0.115
**ORAI1,2-DKO Clone #19**	NA	NA	1.690±0.043

Note: All above values are mean fold change ORAI expression ±SEM. Levels of ORAI 1, ORAI2, or ORAI3 transcript were detected using qPCR and normalized to the housekeeping gene GAPDH (*n*=3 for all conditions).

Legends to Supplemental Figures:

Supplementary Figure 1: STIM1/2, ORAI1 and $IP_3R1/2/3$ isoform expression in ORAI knockout cells

(a, b) Western blot analysis of ORAI1 (a) and STIM1 and STIM2 (b) in parental HEK293 cells and single, double and triple ORAI knockout cells. For the case of ORAI1, whole cell lysates were deglycosylated using PNGase F. When treated with PNGase F two translational variants of ORAI1 are resolved (ORAI1α 32kDa and ORAI1β 25kDa). (c-e) Quantification of ORAI1 (c), STIM1 (d) and STIM2 (e) proteins using densitometry (normalized to GAPDH expression) from several Western blots.

(f) Western blot analysis of IP_3R1, IP_3R2 and IP_3R3 in parental HEK293 cells and single, double and triple ORAI knockout cells. A HEK293 cell line, which lacks all three isoforms of IP_3R has been used side by side as a control for the specificity of IP_3R isoform-specific antibodies. (g-i) Quantification of IP_3R1 (g), IP_3R2 (h) and IP_3R3 (i) proteins using densitometry (normalized to α-tubulin expression) from several Western blots.

Western densitometry ratio data are represented as mean±SEM and were statistically analyzed using a one-way ANOVA with multiple comparisons *p<0.05; ns, not significant). For all conditions in panels c, d, e, and g-i (*n*=3). Each data point for all densitometry data (c-d, g-i) represents an independent experiment.

Supplementary Figure 2: ORAI2/3 isoform expression in ORAI knockout cells

(a) Quantitative RT-PCR documenting ORAI2 and ORAI3 knockout in two clones from each single, double and triple ORAI knockout cell line. All qPCR data was analyzed using Student's t-test (**p<0.01; ***p<0.001; ****p<0.0001; ns, not significant), and all qPCR experiments were run in triplicate (*n*=3 for all conditions). For clones with remaining mRNA see **Supplementary Table 4** for a detailed list of genetic changes; potential compensatory upregulation or downregulation of ORAI mRNA was also addressed in **Supplementary Table 5**.

Supplementary Figure 3: ORAI-mediated CRAC currents are blocked by 5µM Gd^{3+}

(a, d, g) Representative CRAC currents were recorded with a pipette solution containing 20mM BAPTA and elicited by voltage ramps from -140mV to +100mV (from a holding potential of +30mV) from ORAI-TKO cells co-expressing YFP-STIM1 (4µg plasmid) with individual CFP-ORAI isoforms (1 µg plasmid). 5 µM Gd^{3+} was perfused to the bath when current reached maximal steady state.

(b, e, h) Representative I/V curves of maximal CRAC currents and after perfusion of 5 µM Gd^{3+} for ORAI1 (b), ORAI2 (e) and ORAI3 (h). Curves represented are taken from respective traces in (a, d, g) where indicated by color-coded asterisks (Red= after Gd^{3+} and Black= before Gd^{3+}).

(c, f, i) Peak CRAC current densities and remaining current after perfusion of 5 µM Gd^{3+} were taken at -100mV and shown as scatter plots for each ORAI isoform. Each data point represents

mean ± SEM. Data were statistically analyzed using a two-tailed Student's t-test (**p<0.01; ***p<0.0001). Boxplots show the mean, median, and the 75th to 25th percentiles.

Supplementary Figure 4: CRAC currents mediated by ORAI concatenated heterodimers are blocked by 5μM Gd^{3+}

(a, d, g, j) Representative CRAC currents were recorded with a pipette solution containing 20mM BAPTA and elicited by voltage ramps from -140mV to +100mV (from a holding potential of +30mV) from ORAI-TKO cells co-expressing YFP-STIM1 (4μg plasmid) with td-Tomato-tagged ORAI concatenated homo- and heterodimers (1 μg plasmid). 5 μM Gd^{3+} was perfused to the bath when current reached maximal steady state.

(b, e, h, k) Representative I/V curves of maximal CRAC currents and after perfusion of 5 μM Gd^{3+} for ORAI1,1 homodimer (b), ORAI1,2 heterodimer (e) and ORAI1,3 heterodimer (h) and ORAI2,3 heterodimer (j). Curves represented are taken from respective traces in (a, d, g, j) where indicated by color-coded asterisks (Red= after Gd^{3+} and Black= before Gd^{3+}).

(c, f, I, l) Peak CRAC current densities and remaining current after perfusion of 5 μM Gd^{3+} were taken at -100mV and shown as scatter plots for each ORAI concatemer. Each data point represents mean ± SEM. Data were statistically analyzed using a two-tailed Student's t-test (***p<0.001). Boxplots show the mean, median, and the 75th to 25th percentiles.

Supplementary Figure 5: Ca^{2+} oscillations in ORAI1-SKO and ORAI1,3-DKO cells persist for over an hour

(a-d) Representative Ca^{2+} oscillations in response to 10μM carbachol (Cch) measured using Fura2 over the course of 60 min in wildtype HEK293 cells (*n*= 102) (a), ORAI1-SKO cells (*n*= 107) (b), ORAI1,3-DKO cells (*n*= 71) (c) and ORAI-TKO cells (*n*= 53) (d). Cells were maintained in HBSS containing 2mM Ca^{2+} for the duration of the experiments and stimulated with 10μM CCh at 1min (where indicated by arrow in "a"). Representative traces from 5 cells/condition were chosen to represent the datasets as a whole. (e) Quantification of total oscillations/59 min for all conditions from (a-d). Scatter plot in (e) represents mean±SEM and data were statistically analyzed using the Kruskal-Wallis one-way ANOVA with multiple comparisons correction (*p<0.05). All comparisons were made to WT HEK293. Arrow in panel "a" indicates the time of Cch addition.

Supplementary Figure 6: All three ORAI isoforms interact with one another

(a-c) Scatter plot of E-FRET from several cells represented as mean±SEM under basal conditions and after addition of increasing concentrations of Cch (10-100 μM) followed by 1μM ionomycin (Iono) in HBSS containing 2mM Ca^{2+}. E-FRET between CFP-ORAI1/YFP-ORAI2 (*n*= 42) (a), CFP-ORAI1/YFP-ORAI3 (*n*= 39) (b) and CFP-ORAI2/YFP-ORAI3 (*n*= 33) (c) are shown. (d) Scatter blot comparing mean±SEM of baseline E-FRET between the aforementioned three conditions. (e) mean±SEM of CFP/YFP ratios for all cells analyzed in (a-d). All data are represented as mean±SEM and were statistically analyzed using the Kruskal-Wallis one-way ANOVA with multiple comparisons (ns, not significant). In panels (a-c), comparisons were to basal FRET. All possible comparisons were made for data in panels (d-e) and all lacked significance.

Supplementary Figure 7: % of oscillating cells and plateaus cells in ORAI pore mutant-transfected cells

(a-c) % of oscillating cells (a), % of plateaus cells (b) and % of non-responding cells (c) for experiments depicted in Figure 5a-f where parental HEK293 cells and ORAI1-SKO cells were transfected with ORAI pore mutant constructs, namely ORAI1-E106Q, ORAI1-E106A, ORAI2-E80Q and ORAI3-E81Q. All results were analyzed using one-way ANOVA with multiple comparisons (*p<0.05). All comparisons were made between the corresponding untransfected control (i.e WT HEK293 or ORAI1-SKO) (From left to right *n*= 4, 4, 6, 6, 6, 6, 6, 6, 3, and 3; data points represent independent experiments).

Supplementary Figure 8: CRAC currents mediated by ORAI concatenated heterodimers are blocked by 5µM Gd^{3+}

(a, d, g) Representative CRAC currents were recorded with a pipette solution containing 20mM BAPTA and elicited by voltage ramps from -140mV to +100mV (from a holding potential of +30mV) from ORAI-TKO cells co-expressing YFP-STIM1 (4µg plasmid) with individual CFP-ORAI isoforms (1 µg plasmid). 50 µM 2-APB was perfused into the bath when current reached maximal steady state, followed by 5 µM Gd^{3+} where indicated by arrows.

(b, e, h) Representative I/V curves of maximal CRAC currents and after perfusion of 50 µM 2-APB for ORAI1 (b), ORAI2 (e) and ORAI3 (h). Curves represented are taken from respective traces in (a, d, g) where indicated by color-coded asterisks (Red= after 2-APB and Black= before 2-APB).

(c, f, i) Peak CRAC current densities before and after perfusion of 50 µM 2-APB were taken at -100mV and shown as scatter plots for each ORAI isoform. Each data point represents mean ± SEM. Data were statistically analyzed using a two-tailed Student's t-test (**p<0.01). Boxplots show the mean, median, and the 75th to 25th percentiles.

Supplementary Figure 9: ORAI-mediated CRAC currents are inhibited by 10 µM GSK-7975A

(a, d, g) Representative CRAC currents were recorded with a pipette solution containing 20 mM BAPTA and membrane currents were elicited by voltage ramps from -140 mV to +100 mV (from a holding potential of +30 mV) from ORAI-TKO cells co-expressing YFP-STIM1 (4 µg plasmid) with individual CFP-ORAI isoforms (1 µg plasmid). 10 µM GSK-7975A was perfused in the bath when current reached maximal steady state.

(b, e, h) Representative I/V curves of maximal CRAC currents before and after perfusion of 10 µM GSK-7975A for ORAI1 (b), ORAI2 (e) and ORAI3 (h). Curves represented are taken from respective traces in (a, d, g) where indicated by color-coded asterisks (Red= after GSK and Black= before GSK).

(c, f, i) Peak CRAC current densities and remaining currents after perfusion of 10 µM GSK-7975A were taken at -100 mV and shown as scatter plots for each ORAI isoform. Each data

point represents mean ± SEM. Data were statistically analyzed using two-tailed Student's t-test (***, $p<0.005$). Boxplots show the mean, median, and the 75th to 25th percentiles.

Supplementary Figure 10: ER Ca^{2+} concentrations in response to agonist stimulation

Model simulations used the parameter p (with units of µM) as the equivalent to increasing agonist concentrations. Other parameter values are $I_1 = 1$, $I_2 = 0.2$, $I_3 = 0.2$ (all in units of concentration), $K_{11} = 14$, $K_{22} = 1.5$, $K_{33} = 0.93$, $K_{12} = 19$, $K_{13} = 17$, $K_{23} = 14$ (all in units of 1/concentration), $\alpha_{11} = 2$, $\alpha_{22} = 1$, $\alpha_{33} = 0.7$, $\alpha_{12} = 0.1$, $\alpha_{13} = 0.1$, $\alpha_{23} = 0.1$ (all in units of 1/time). All other parameter values are as in[22]. (a-c) model the ER Ca^{2+} response to increasing agonist concentrations in parental HEK293 cells and 5 different single, double, and triple ORAI knockout cell lines. (d) Models of the ER Ca^{2+} concentration in response to maximal store depletion with thapsigargin in the absence then presence of 2mM external Ca^{2+}.

Supplementary Figure 11: Representative oscillatory responses

Three traces representing the three oscillatory profiles quantified throughout the manuscript. Oscillating cells are cells that display regenerative oscillations for the duration of the experiment, where each oscillation returns to baseline before the start of the next oscillation. Non-responders are cells that either do not respond at all to agonist or show only one initial spike that returned to baseline. Plateau cells are cells that respond in the form of sustained elevation of cytosolic Ca^{2+}, ≥25% of the initial peak, for at least 5min post agonist stimulation. The arrow at ~1min on the x-axis indicates the addition of 10µM Cch to the bath solution.

Supplementary Figure 12: Number of combinations of ORAI heterohexamers

According to Burnside's Lemma, the number of distinct 3-colorings (3 ORAI isoforms) within a hexagon (hexameric CRAC channels) corresponds to the six rotational symmetries of the 6-positions within the hexagon (π); and the six reflective symmetries of the hexagon (r).

Supplementary Figure 13: ORAI proteins localize to the plasma membrane

(a) Representative confocal images of ORAI-TKO cells transiently transfected with either YFP-ORAI1, YFP-ORAI2, or YFP-ORAI3. (b) Representative confocal images of ORAI-TKO cells transiently transfected with either ORAI1,2-tdTomato, ORAI1,3-tdTomato, or ORAI2,3-tdTomato. (a-b) Scale bar = 10µm. These experiments were repeated at least 4 times with similar results.

Supplementary Figure 1

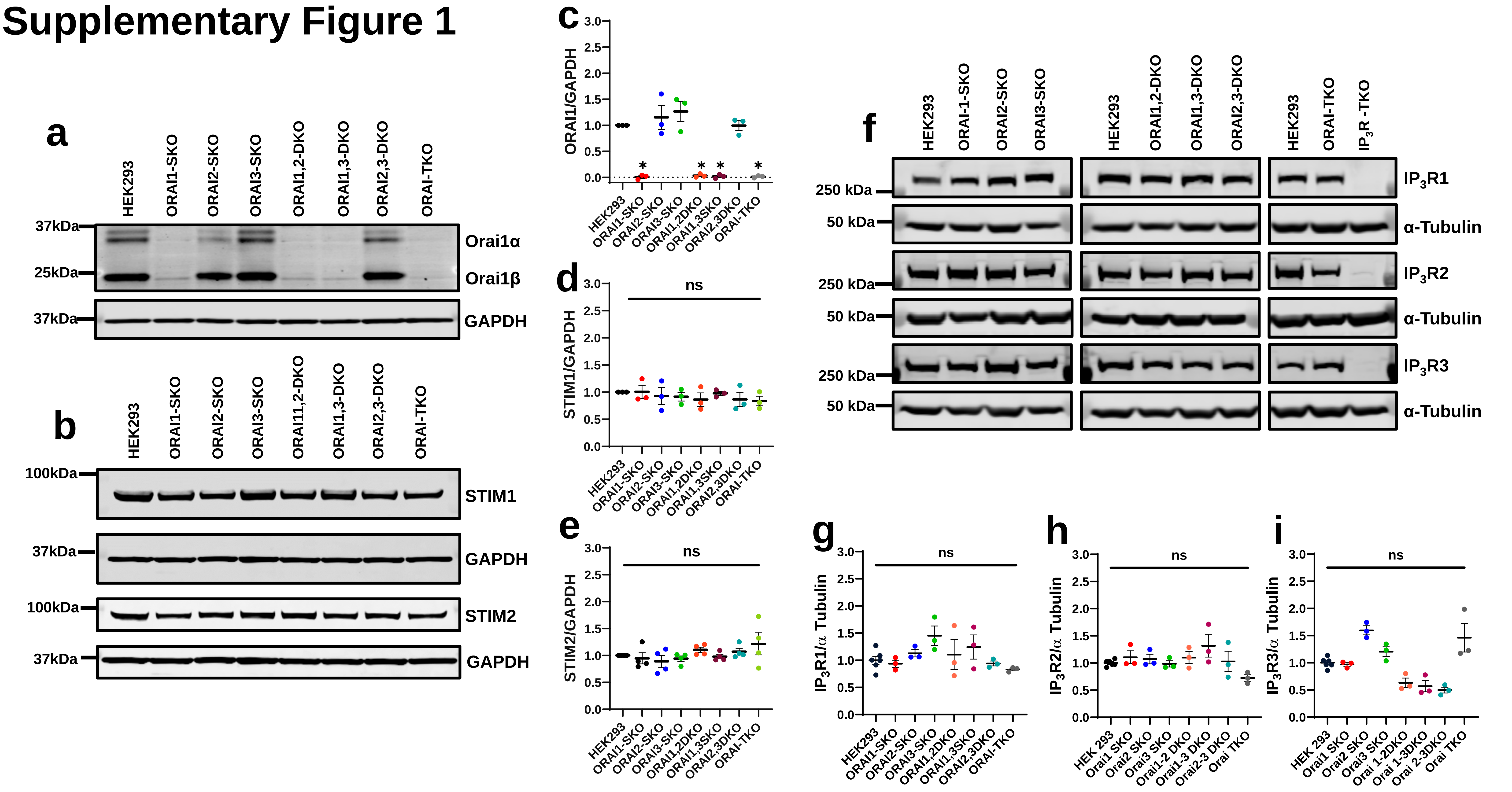

Supplementary Figure 2

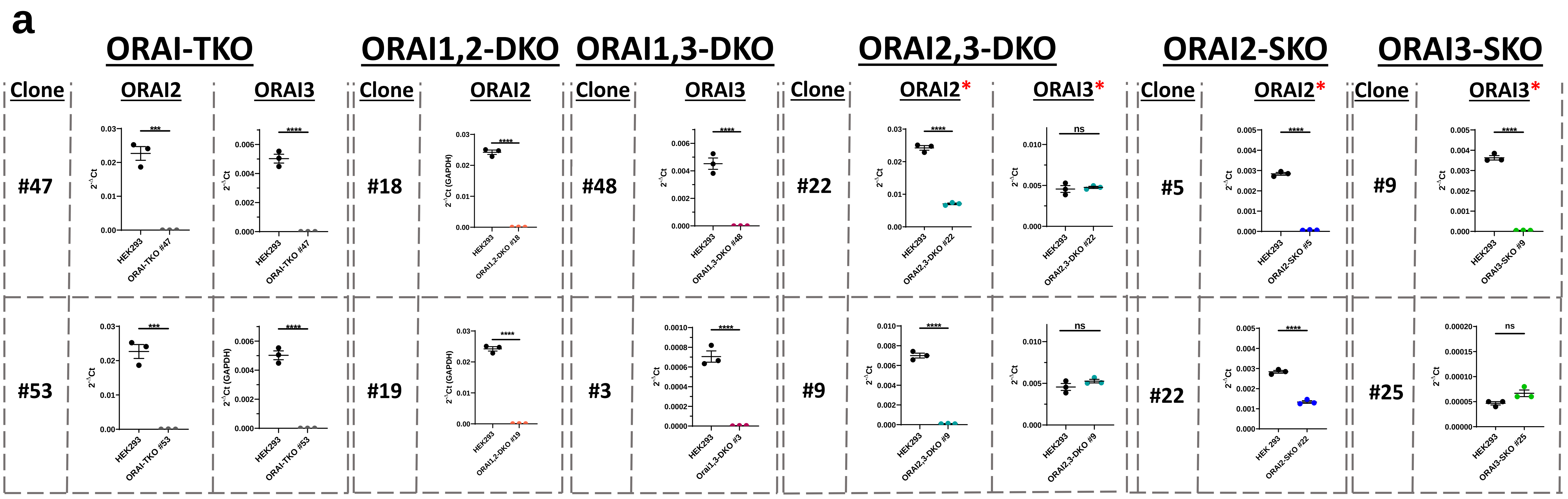

*indicates alternative lines of evidence validating ORAI knockout (see supplementary table 4)

Supplementary Figure 3

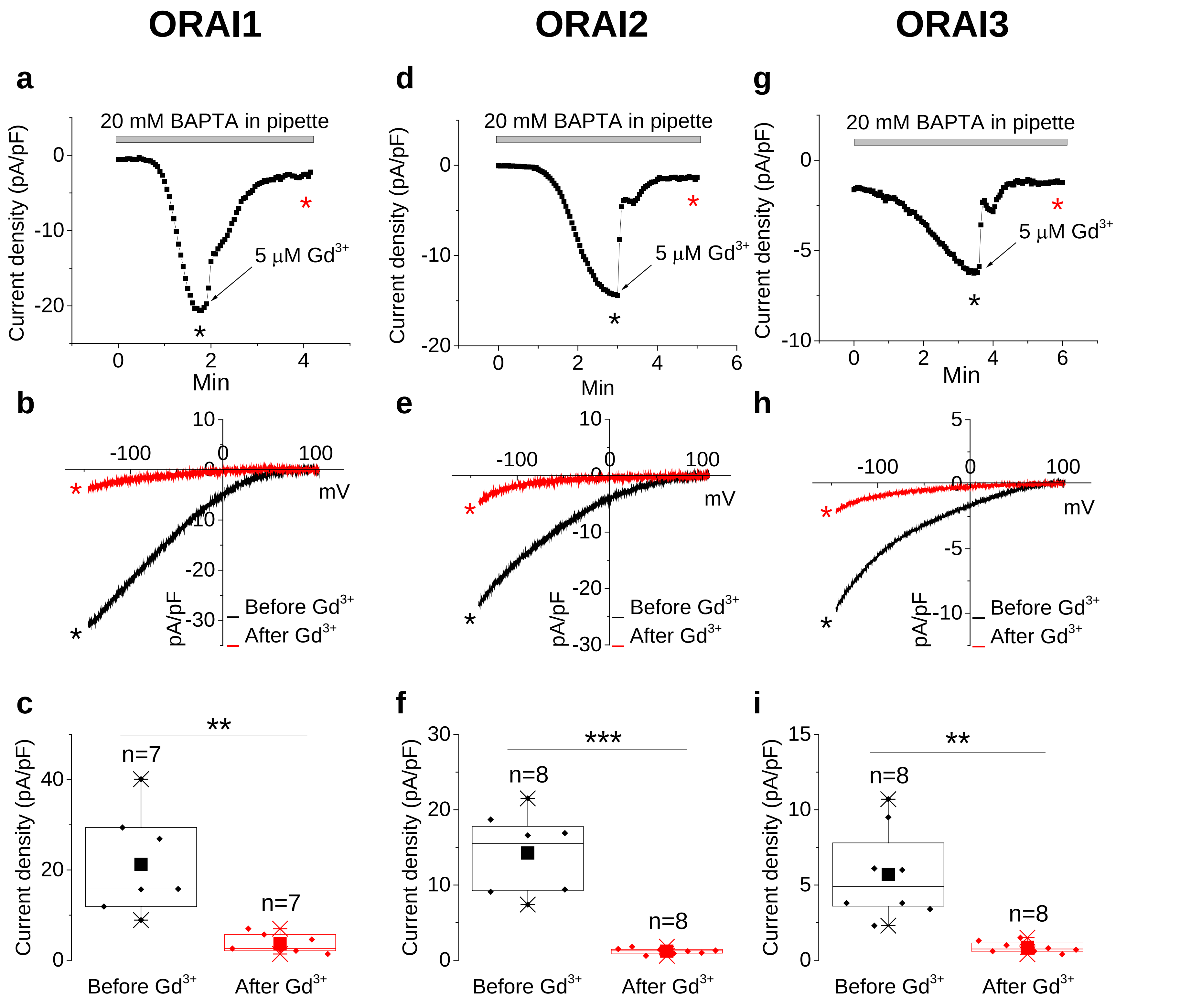

Supplementary Figure 4

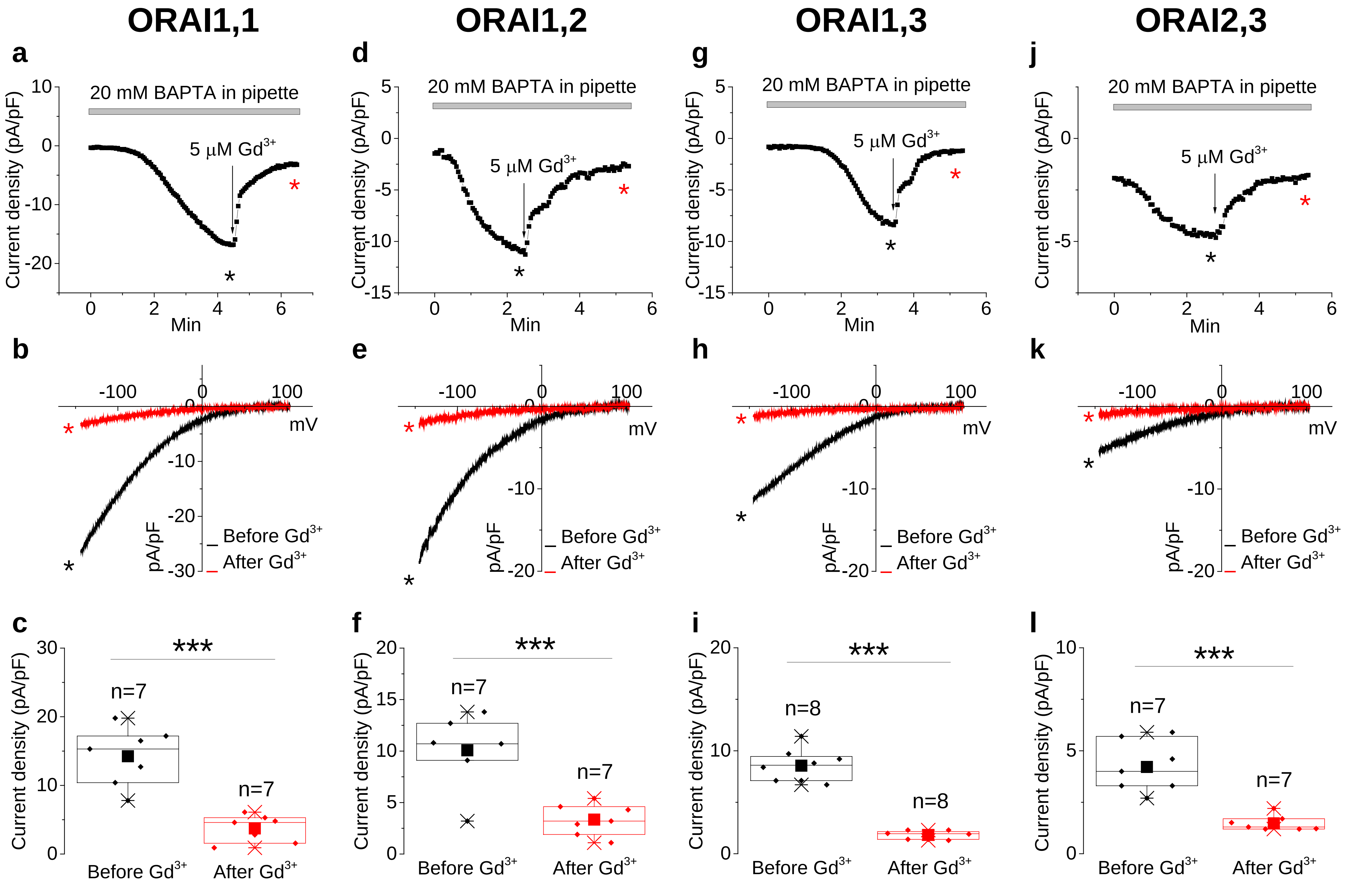

Supplementary Figure 5

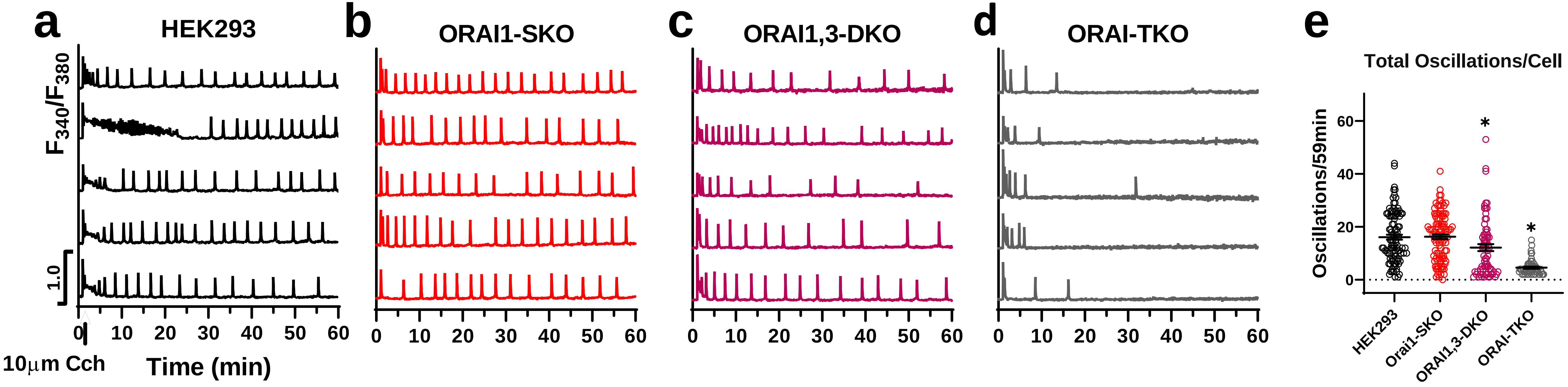

Supplementary Figure 6

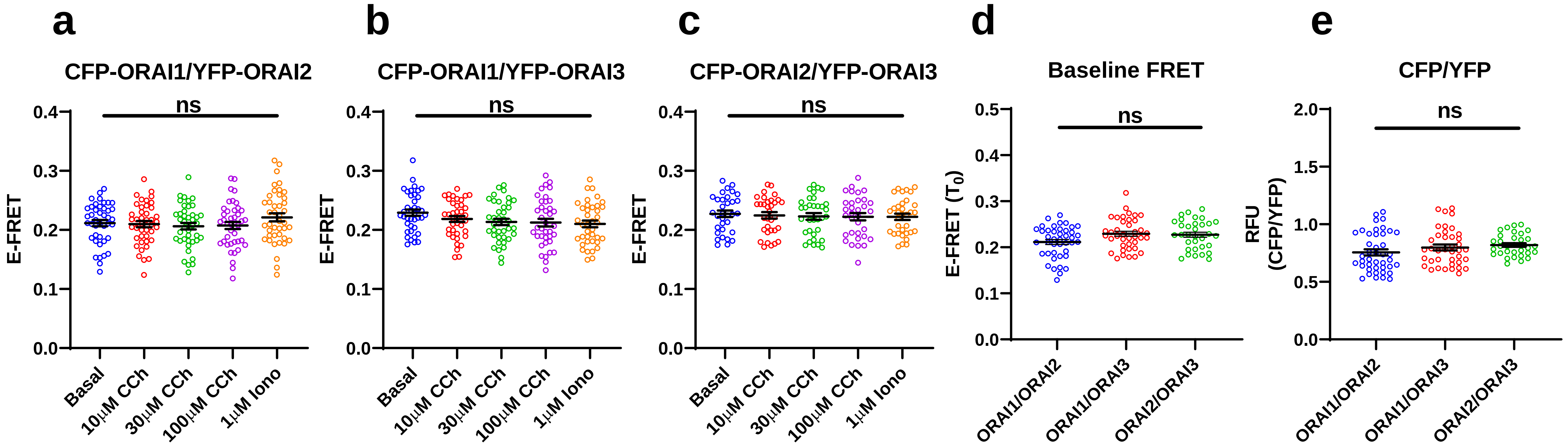

Supplementary Figure 7

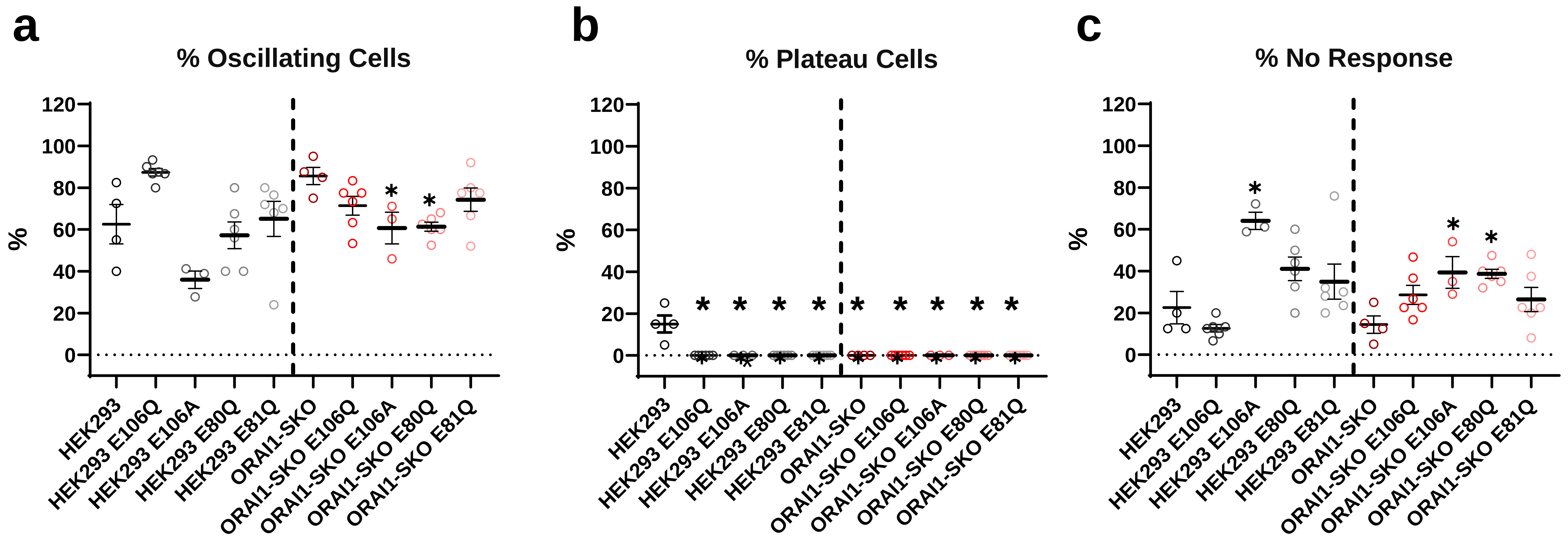

Supplementary Figure 8

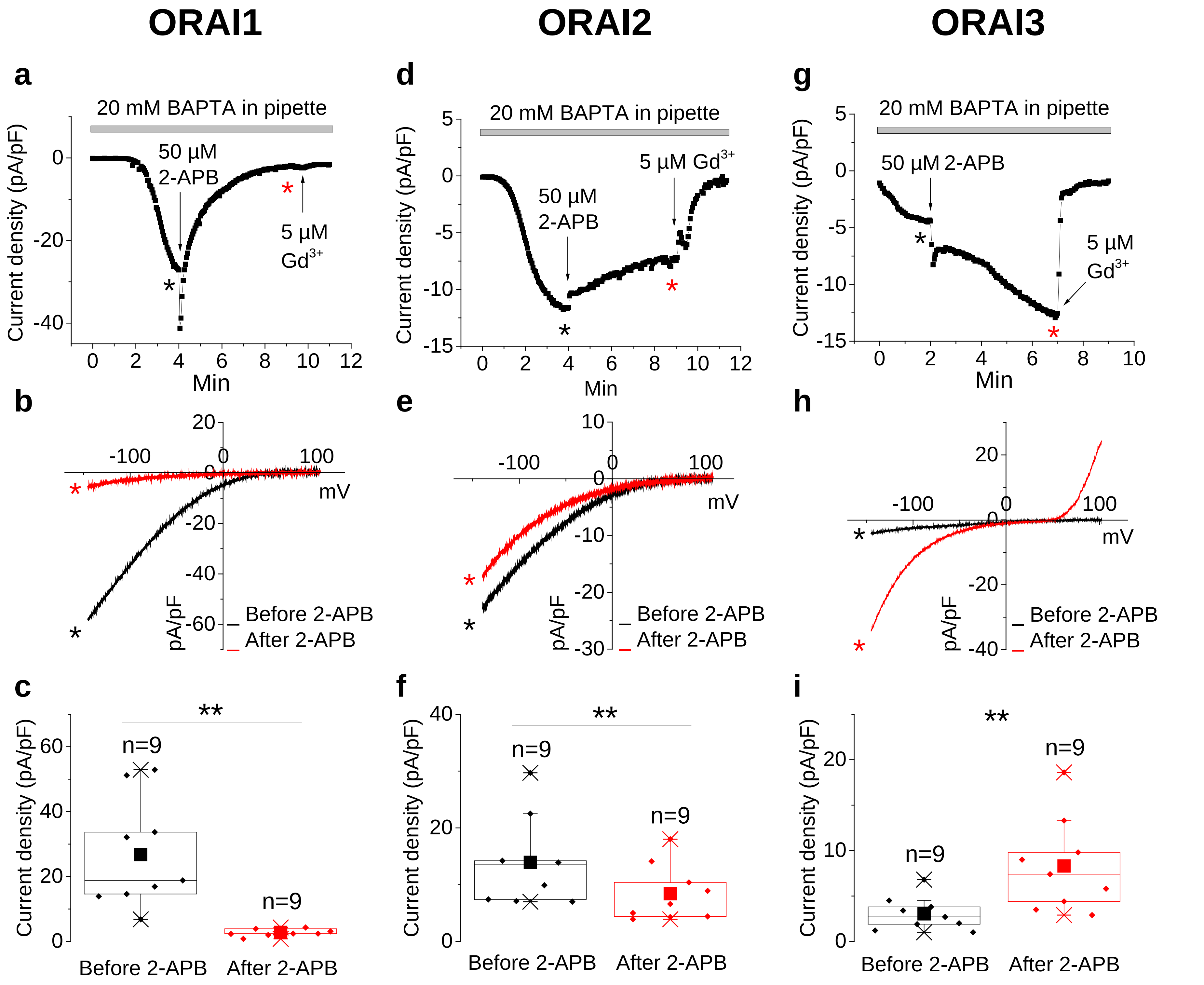

Supplementary Figure 9

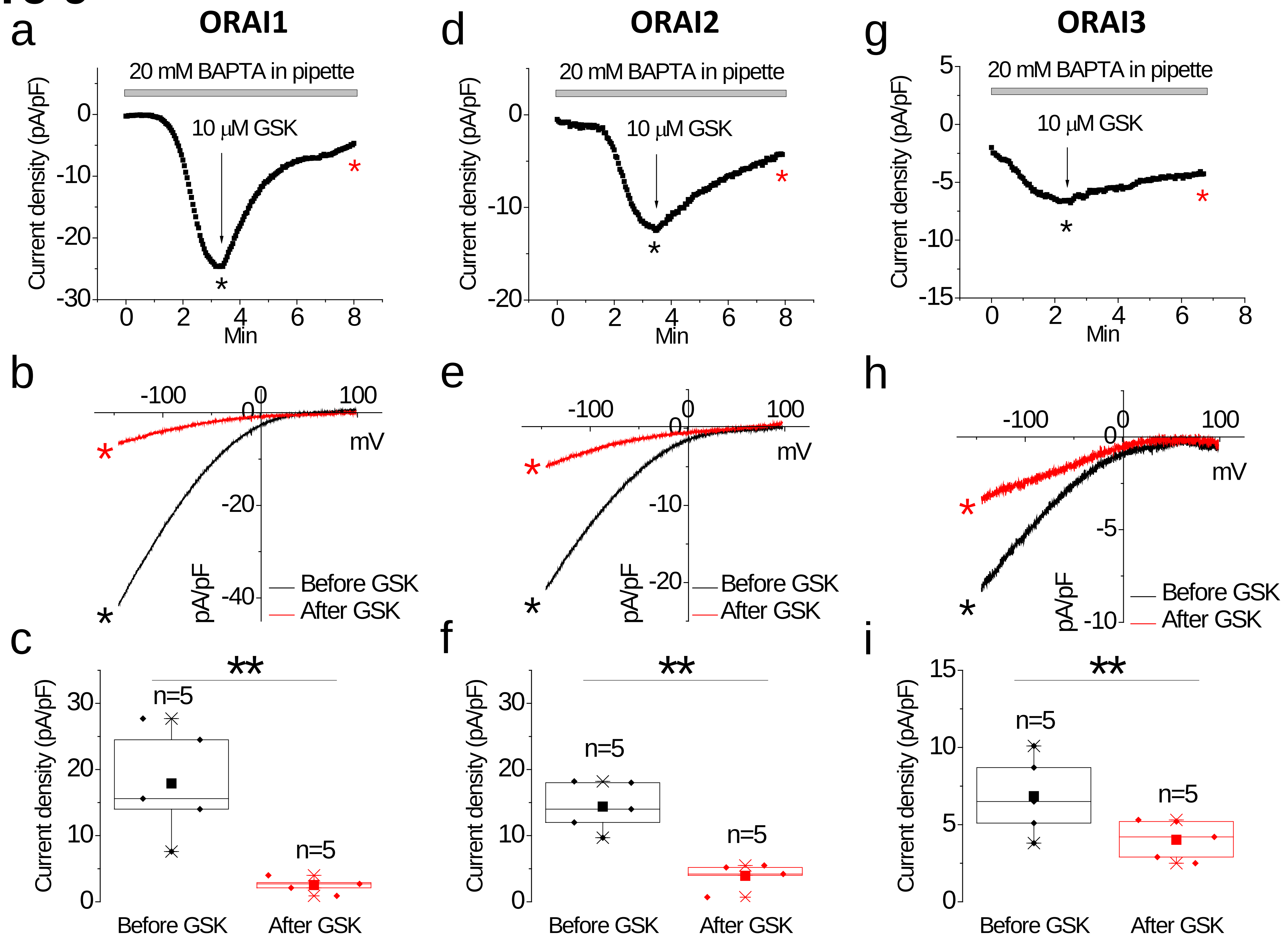

Supplementary Figure 10

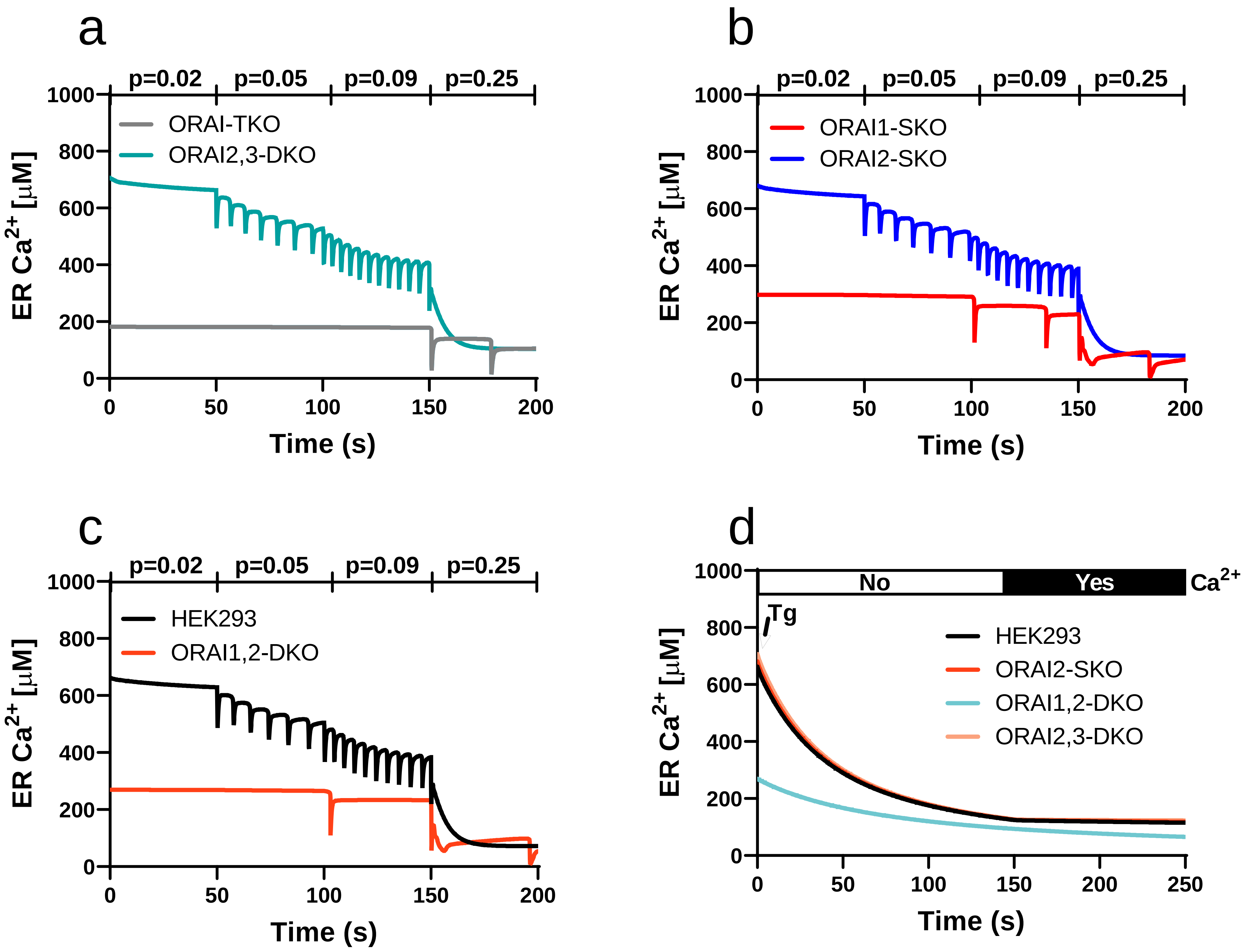

Supplementary Figure 11

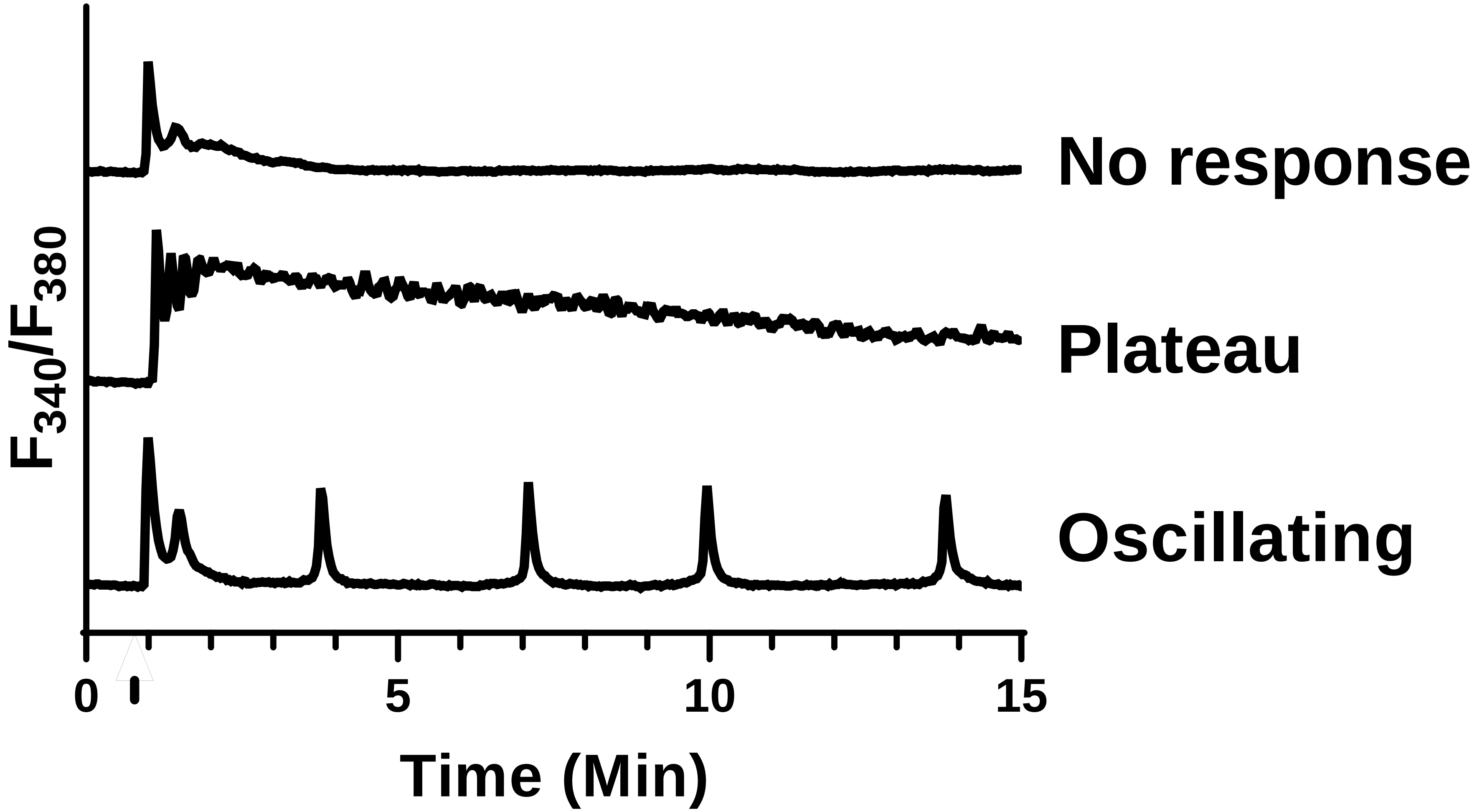

Supplementary Figure 12

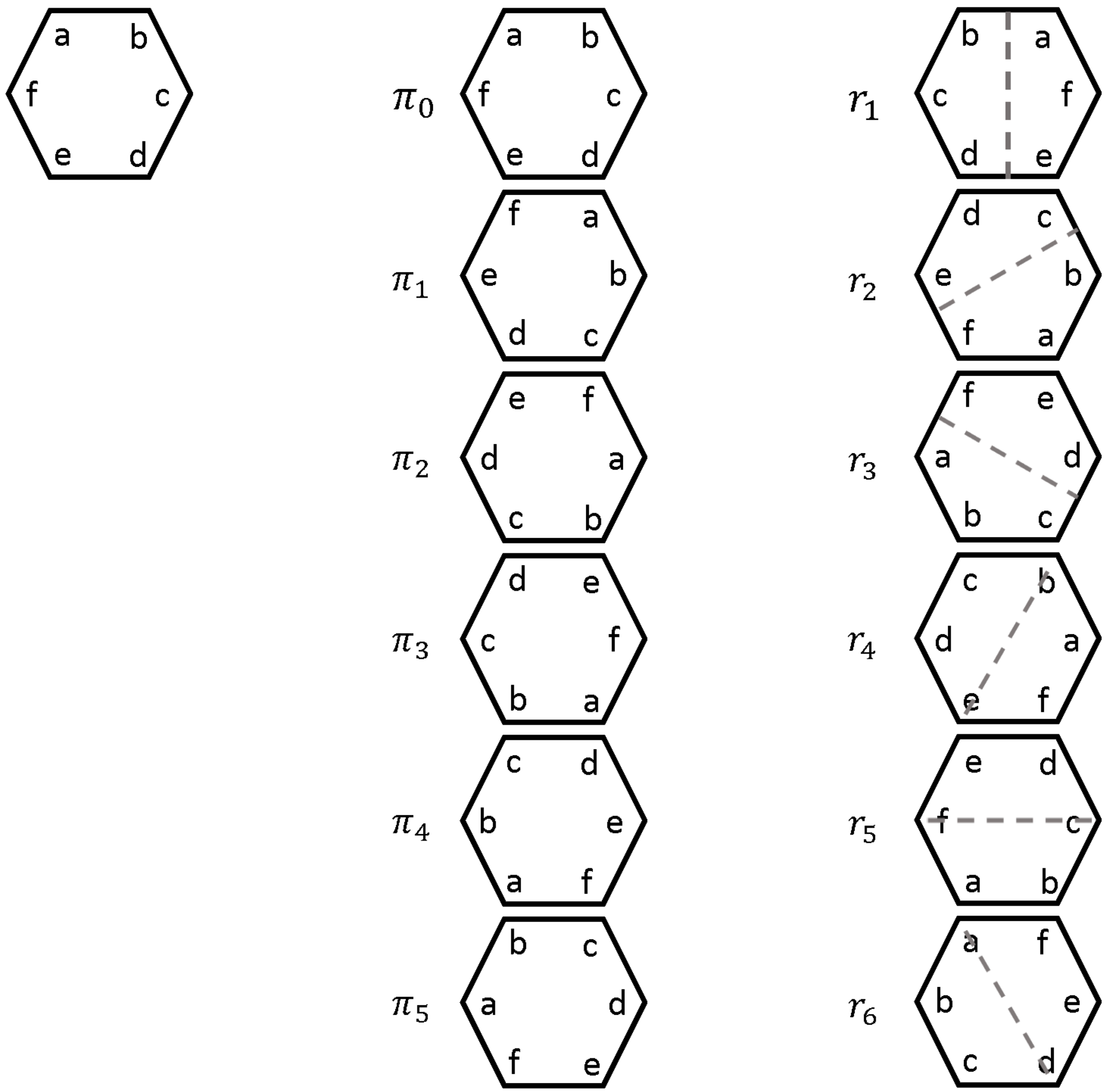

Supplementary Figure 13

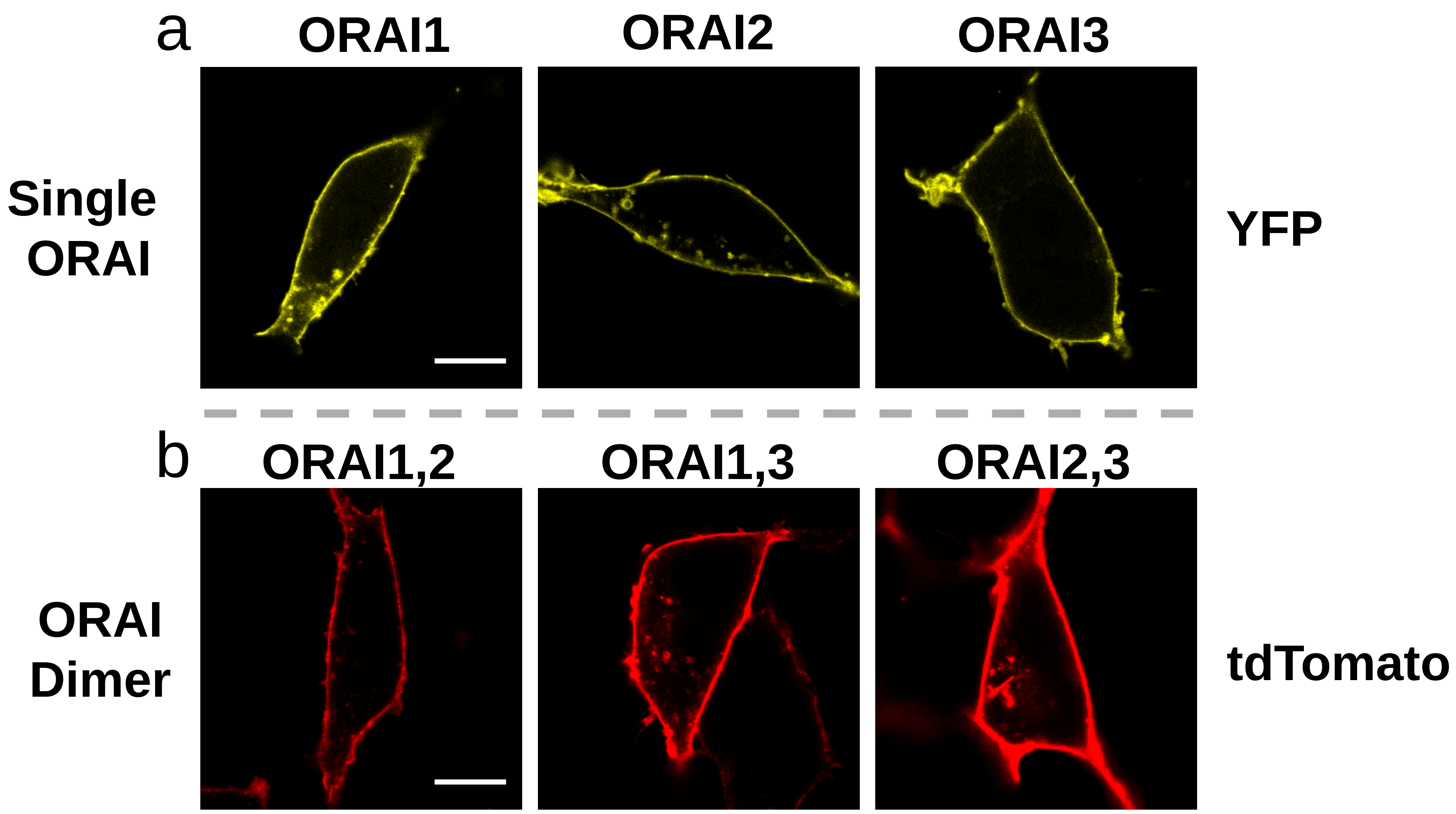

Supplement: Supplementary file 1 — Supplementary Information [file 41467_2020_16232_MOESM1_ESM.pdf]
