# Supplementary material for: The native ORAI channel trio underlies the diversity of Ca2+ signaling events
Source: Nat Commun. 2020 May 15;11:2444. doi: 10.1038/s41467-020-16232-6 (PMC7229178; doi:10.1038/s41467-020-16232-6)
Supplement: Supplementary file 3 — Description of Additional Supplementary Files [file 41467_2020_16232_MOESM3_ESM.pdf]

Description of Additional Supplementary Files

File name: Supplementary Data 1

Description: List of "p values" of all statistical comparisons performed in the study
